# Supplementary material for: Tuning Localized Surface Plasmon Resonance of Nanoporous Gold with a Silica Shell for Surface Enhanced Raman Scattering
Source: Nanomaterials (Basel). 2019 Feb 12;9(2):251. doi: 10.3390/nano9020251 (PMC6410204; doi:10.3390/nano9020251)
Supplement: Supplementary file 1 [file nanomaterials-09-00251-s001.pdf]

Tuning localized surface plasmon resonance of nanoporous gold with silica shell for surface enhanced Raman scattering

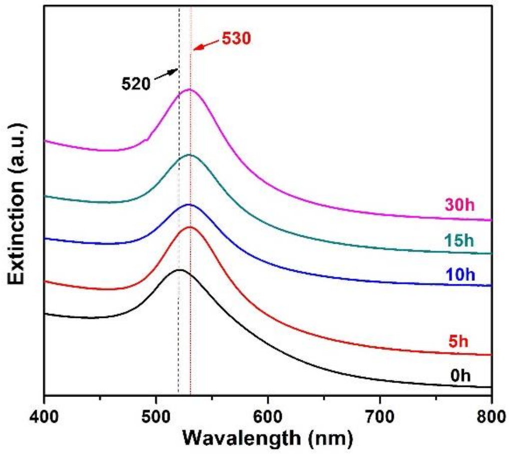

Figure. S1. Extinction spectra of SiO_2@AuNPs with different silica coating time (0h represents bare AuNPs).

The extinction spectral features of 10nm AuNPs and SiO_2@AuNPs with different silica shell thicknesses shown in Fig. S1. The characteristic bands of SiO_2@AuNPs are quite similar to that of AuNPs with a slight shift to the red, indicating the effect of the shell on the localized surface plasmon resonance (LSPR) is negligible [26].
